# Analytical study on ignition time of PMMA exposed to time-decreasing thermal radiation using critical mass flux

**DOI:** 10.1038/s41598-019-48411-x

**Published:** 2019-08-16

**Authors:** Chunjie Zhai, Siyu Zhang, Shuren Yao, Qingbin Zhan, Shuifeng Zhang, Yue Wang

**Affiliations:** 1 Department of Information Technology, Nanjing Forest Police College, Nanjing, Jiangsu 210023 China; 2 College of Safety Science and Engineering, Nanjing Tech University, Nanjing, Jiangsu 210009 China; 3 Engineering Research Center of Forest Fire Management, State Forestry and Grassland Administration, Nanjing, Jiangsu 210023 China

**Keywords:** Mechanical engineering, Computational methods

## Abstract

This contribution addresses an analytical model to predict the ignition time of PMMA (Polymethyl methacrylate) subjected to a time-decreasing incident heat flux. Surface temperature, transient mass flux and ignition time of PMMA are thoroughly studied based on the exact solutions of in-depth temperature. Critical mass flux is utilized as the ignition criteria. An approximation methodology is suggested to simplify the unsolvable high order equations and deduce the explicit expressions of ignition time. A numerical model is employed to validate the capability of the developed model. The results show that no ignition occurs when the decreasing rate of heat flux increases larger than a critical value. The agreement of the transient mass flux between analytical and numerical models is good at high decreasing rate but turns worse as the decreasing rate declines. However, this enhanced discrepancy affects the ignition time prediction slightly. The inverse of the square root of the ignition time is linearly correlated with the decreasing rate of heat flux, and it becomes significantly sensitive to the decreasing rate when the decreasing rate approaching its critical value. Meanwhile, the value of critical mass flux has appreciable influence on the ignition time prediction.

## Introduction

Pyrolysis and the subsequent ignition under external incident heat flux (HF) are important for fire protection since they determine the occurrence of fire propagation and are easier to be controlled at early stage. Ignition under constant^1–8^ and time-increasing^9–19^ HFs gained much attention and have been studied extensively in the literature, including empirical models^1,5,6,13,15^, analytical^4,7–10,12,17,18^ and numerical models^2,3,11,20–23^. However, the time-decreasing HF, which may result from a moving-away flame in forest fire, high temperature smoke during cooling process in compartment fire before flashover or irradiative heater after power outage^24^, is barely concerned and the ignition behaviors of the imposed materials are not adequately understood.

Under constant HF, the pioneers did some remarkable works to reveal the ignition mechanism, such as the classical ignition theory^1,25^ in which pyrolysis and the dependence of thermal parameters on temperature were neglected. Critical temperature was used in these models. Based on this original theory, other researchers improved the model when dealing with some influential aspects, such as the surface heat loss^26,27^, porosity of materials and the mass transfer of the yielded gas in solid^28–31^, grain orientation of wood^32^, ambient pressure and oxygen concentration^30,31,33–35^, air flow velocity on surface^36^, generated char layer^37^, melting behaviors^38^, gravity^39^, in-depth absorption of thermal radiation^2–4,7^, optical and radiative properties of semitransparent materials^40,41^. In order to introduce the critical mass flux into analytical model, Lautenberger^8^ and Snegirev^42^ used a power law function and Frank-Kamenetskii decomposition^43^ respectively to replace the Arrhenius pyrolysis rate and derived explicit expressions for ignition time. Some remarkable works aiming at providing insight into the thermal degradation of solid utilizing multi-component Arrhenius expressions were implemented through TGA, DSC, MCC and cone calorimetry^44–47^. Constant HF was applied in these studies owing to the fact that this condition is comparatively easier to conduct using standard experimental apparatus at bench scale tests. However, the materials received HF frequently varies with a propagating flame, growth or decline of fires.

While under time-increasing HF, Lamorlette^18^ discussed an approach to determine the analytical solutions, including power law and polynomial HFs. Didomizio^10^ experimentally and numerically studied the ignition of wood under fourth-order HF. Vermesi^12,13^ investigated the ignition of PMMA and wood under parabolic HF by the FPA. Also, a numerical solver Gpyro^21^ was employed to implement the simulation. Yang^14^ and Ji^15^ designed a linearly increasing HF in their tests to explore the ignition of wood species. An integral model was developed to analyze the experimental data. Later, Zhai^17^ extended Yang and Ji’s works to exponential HF. In all these studies, critical temperature and critical mass flux were commonly used for analytical and numerical models, respectively. Reszka^9^ studied the ignition time of solid under time-dependent HF in forest fire, and found that the ignition time can be correlated with the total absorbed energy before ignition. Nonetheless, critical mass flux which is believed to be a more reasonable ignition criterion is barely studied under the time-varying HF in these models. Bilbao^24^ examined the ignition behaviors of wood under time-decreasing HF in his experiment by turning off the power supply of the heater once the predetermined initial HF was reached. A numerical model was introduced in that study to estimate the ignition time. However, no analytical work was provided and the detailed information of the decreasing heat flux was avoided by using an average heat flux before ignition.

In this study, the ignition of PMMA under linearly and quadratically decreasing HFs is investigated analytically. Critical mass flux is utilized to obtain an explicit expression for ignition time with consideration of pyrolysis within solid. Also, transient mass flux is studied in this work. A previously developed numerical model^23^ is employed to validate the proposed model. The capability of the numerical model has been validated through experimental measurements, including the surface temperatures and mass loss rates of several charring and non-charring polymers under constant incident heat flux, and good agreement is found. Furthermore, parametric study is implemented to analyze the dependence of ignition time prediction on ignition criterion.

## Theoretical Analysis

**Figure 1:**
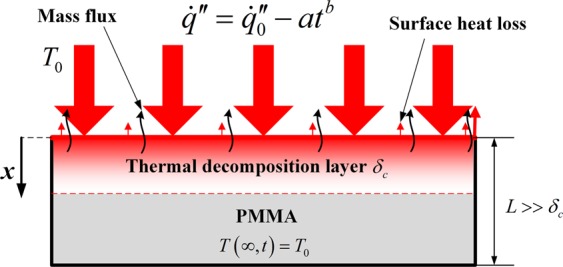
Schematic of heat and mass transfer in PMMA exposed to a time-decreasing HF.

Considering a thermally thick PMMA imposed to a time-decreasing HF, the one-dimensional heat transfer in solid is illustrated in Fig. 1. Relatively low HF, lower than 80 kW/m^2^ ^4^, is focused in this study and only surface absorption of radiation, corresponding to opaque materials, is utilized instead of in-depth absorption. This is because Jiang^7^, Vermesi^12^, Beaulieu^5^, Bal^3,41^, Delichatsios^4^ and Boulet^40^ found surface absorption dominates the heat absorption process even for translucent materials under low HF, whereas in-depth absorption plays a more important role as HF increases. Surface heat loss including convection and reradiation is neglected in this study for simplification. The time-decreasing HF is expressed as:

1 $$\dot{q^{\prime\prime} }={\dot{q^{\prime\prime} }}_{0}-a{t}^{b}$$

where $\dot{q^{\prime\prime} }$ is the transient HF, ${\dot{q}^{\prime\prime} }_{0}$ is the initial HF, *a* and *b* are rational constants. Defining a relative temperature:

2 $$\theta =T-{T}_{0}$$

where *T* and *T*_0_ are the transient and initial temperatures, respectively. The energy conservation equation in solid, initial and boundary conditions can be written as:

3 $$\{\begin{array}{l}\frac{\partial \theta }{\partial t}=\alpha \frac{{\partial }^{2}\theta }{\partial {x}^{2}}\\ \theta (x,0)=0\\ {-k\frac{\partial \theta }{\partial x}|}_{x=0}={\dot{q^{\prime\prime} }}_{0}-a{t}^{b}\\ \theta (\infty ,t)=0\end{array}$$

where *t* is time, *α* is the thermal diffusivity, $\alpha =k/\rho {C}_{p}$, *k* is thermal conductivity, $\rho$ is the density, *C*_*p*_ is the specific heat and *x* is the spatial variable. Equation (3) can be further decomposed as:

4 $$\theta ={\theta }_{1}-{\theta }_{2}$$

5 $$\{\begin{array}{l}\frac{\partial {\theta }_{1}}{\partial t}=\alpha \frac{{\partial }^{2}{\theta }_{1}}{\partial {x}^{2}}\\ {\theta }_{1}(x,0)=0\\ {-k\frac{\partial {\theta }_{1}}{\partial x}|}_{x=0}={\dot{q^{\prime\prime} }}_{0}\\ {\theta }_{1}(\infty ,t)=0\end{array}\,\{\begin{array}{l}\frac{\partial {\theta }_{2}}{\partial t}=\alpha \frac{{\partial }^{2}{\theta }_{2}}{\partial {x}^{2}}\\ {\theta }_{2}(x,0)=0\\ {-k\frac{\partial {\theta }_{2}}{\partial x}|}_{x=0}=a{t}^{b}\\ {\theta }_{2}(\infty ,t)=0\end{array}$$

*θ*_1_ and *θ*_2_ are the solutions of constant and time-increasing HF scenarios, respectively. *θ*_2_ was derived in ref.^19^. *θ*_1_ is equivalent to *θ*_2_ when $a={\dot{q^{\prime\prime} }}_{0}$, $b=0$. Consequently, the solution of *θ* can be obtained as:

6 $$\theta =\frac{2\sqrt{t}{\dot{q^{\prime\prime} }}_{0}}{\sqrt{k\rho {C}_{P}}}ierfc(\frac{x}{2\sqrt{\alpha t}})-\frac{ab!}{\sqrt{k\rho {C}_{P}}}{(4t)}^{(2b+1)/2}{i}^{2b+1}erfc(\frac{x}{2\sqrt{\alpha t}})$$

After defining a dimensionless variable $\xi =x/{\delta }_{c}$ where ${\delta }_{c}=2\sqrt{\alpha t}$, Eq. (6) can be rewritten as:

7 $$\theta (\xi ,t)=\frac{2\sqrt{t}{\dot{q^{\prime\prime} }}_{0}}{\sqrt{k\rho {C}_{P}}}ierfc(\xi )-\frac{ab!}{\sqrt{k\rho {C}_{P}}}{(4t)}^{(2b+1)/2}{i}^{2b+1}erfc(\xi )$$

Equation (6) or (7) is the transient in-depth temperature in solid. Some researchers tried to integrate the pyrolysis reaction into analytical models by invoking some approximations. For instance, Lautenberger^8^ replaced the pyrolysis rate by a power law function, and Snegirev^42^ used the Frank-Kamenetskii decomposition to simplify the Arrhenius function. However, all these works were conducted under constant HF. In this section, we extend Lautenberger’s^8^ study from constant to time-decreasing boundary condition. Both linearly and quadratically decreasing HFs are examined.

### Linearly decreasing heat flux

The total transient mass flux of a thermally thick solid subjected to an incident HF can be expressed as^19^:

8 $$\dot{m}^{\prime\prime} =\rho Z\,{\int }_{0}^{\infty }\,exp(\,-\,{T}_{a}/T)dx\approx \rho Z{\delta }_{c}\,{\int }_{0}^{1}\,exp(\,-\,{T}_{a}/T)d\xi $$

where $\dot{m}^{\prime\prime}$ is mass flux, *Z* is the pre-exponential factor and *T*_*a*_ is the activation temperature. As the approximation results in ref.^8^, the pyrolysis rate can be replaced by a power law function when the pyrolysis temperature is between 250 °C and 450 °C and the *T*_*a*_ ranges from 10000 K to 30000 K:

9 $$\exp (\,-\frac{{T}_{a}}{T})\approx A{(\frac{T}{{T}_{0}})}^{B}$$

10 $$A=\exp (\,-\frac{{T}_{a}}{{T}_{1}});{T}_{1}\approx 357\,K$$

11 $$B={T}_{a}/{T}_{2};{T}_{2}\approx 615\,K$$

where *A* and *B* are constants, *T*_*1*_ and *T*_*2*_ are characteristic temperatures. For linearly decreasing HF, *b* = 1, Eq. (7) can be rewritten as:

12 $$\theta =\frac{2\sqrt{t}{\dot{q^{\prime\prime} }}_{0}}{\sqrt{k\rho {C}_{P}}}ierfc(\xi )-\frac{a}{\sqrt{k\rho {C}_{P}}}{(4t)}^{3/2}{i}^{3}erfc(\xi )$$

Noticing that

13 $$ierfc(\xi )=\frac{1}{\sqrt{\pi }}{e}^{-{\xi }^{2}}-\xi erfc(\xi )$$

14 $${i}^{3}erfc(\xi )=\frac{1+{\xi }^{2}}{6\sqrt{\pi }}{e}^{-{\xi }^{2}}-\frac{1}{4}(\xi +\frac{2}{3}{\xi }^{3})erfc(\xi )$$

Rearranging Eqs (12–14) and (9), the total mass flux at linearly decreasing HF condition, Eq. (8) can be expressed as:

15 $$\begin{array}{rcl}\dot{m}^{\prime\prime}  & = & \rho Z{\delta }_{c}A\,{\int }_{0}^{1}\,\{1+\frac{2{\dot{q^{\prime\prime} }}_{0}\sqrt{t}}{{T}_{0}\sqrt{k\rho {C}_{P}}}[\frac{1}{\sqrt{\pi }}{e}^{-{\xi }^{2}}-\xi erfc(\xi )]\\  &  & {-\frac{2a{t}^{3/2}}{{T}_{0}\sqrt{k\rho {C}_{P}}}[\frac{2(1+{\xi }^{2})}{3\sqrt{\pi }}{e}^{-{\xi }^{2}}-(\xi +\frac{2}{3}{\xi }^{3})erfc(\xi )]\}}^{B}d\xi \end{array}$$

In order to further simplify Eq. (15), the same exponential approximation in ref.^19^ where the excellent accuracy of this approximation was verified, is utilized as follow:

16 $$\frac{1}{\sqrt{\pi }}{e}^{-{\xi }^{2}}-\xi erfc(\xi )\approx \frac{1}{\sqrt{\pi }}{e}^{-2.3{\xi }^{1.1}}$$

17 $$\frac{2(1+{\xi }^{2})}{3\sqrt{\pi }}{e}^{-{\xi }^{2}}-(\xi +\frac{2}{3}{\xi }^{3})erfc(\xi )\approx \frac{2}{3\sqrt{\pi }}{e}^{-3.25{\xi }^{1.1}}$$

Combining Eqs (16) and (17), Eq. (15) can be rewritten as:

18 $$\dot{m}^{\prime\prime} =\rho Z{\delta }_{c}A\,{{\int }_{0}^{1}[1+\frac{2\sqrt{t}}{{T}_{0}\sqrt{\pi k\rho {C}_{P}}}({\dot{q^{\prime\prime} }}_{0}{e}^{-2.3{\xi }^{1.1}}-\frac{2}{3}at{e}^{-3.25{\xi }^{1.1}})]}^{B}d\xi $$

Unfortunately, Eq. (18) cannot be integrated analytically. Another approximation is introduced to simplify Eq. (18):

19 $$\dot{m}^{\prime\prime} \approx \rho Z{\delta }_{c}A\,{{\int }_{0}^{1}\{[1+\frac{2\sqrt{t}}{{T}_{0}\sqrt{\pi k\rho {C}_{P}}}({\dot{q^{\prime\prime} }}_{0}-\frac{2}{3}at)]{e}^{-\xi }\}}^{B}d\xi $$

**Figure 2:**
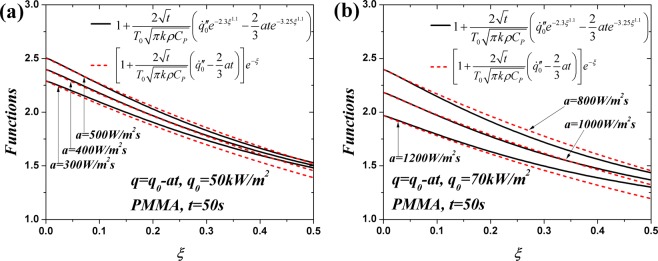
Comparison of the expressions in the square brackets of Eqs (18) and (19).

The reliability of this assumption is validated in Fig. 2. Although the approximate curves deviate more greatly as $\xi$ increases, they agree well with the exact solutions near the high temperature surface where the pyrolysis reaction contributes the most of the mass flux. Accordingly, the transient mass flux can be expressed as:

20 $$\dot{m}^{\prime\prime} =\rho Z{\delta }_{c}A{C}_{1}{[1+\frac{2\sqrt{t}}{{T}_{0}\sqrt{\pi k\rho {C}_{P}}}({\dot{q^{\prime\prime} }}_{0}-\frac{2}{3}at)]}^{B}$$

21 $${C}_{1}=(1-{e}^{-B})/B$$

Ignition occurs only if the maximum mass flux is greater than the critical mass flux, ${\dot{m}^{\prime\prime} }_{\max ,1}\ge {\dot{m}^{\prime\prime} }_{cri}$. Based on Eq. (20), it can be derived that:

22 $$\frac{2\sqrt{{t}_{\max ,1}}}{{T}_{0}\sqrt{\pi k\rho {C}_{P}}}({\dot{q^{\prime\prime} }}_{0}-\frac{2}{3}a{t}_{\max ,1})\ge {\theta }_{ig,1}^{\ast };\,{\theta }_{ig,1}^{\ast }={T}_{ig,1}^{\ast }-{T}_{0}$$

23 $${T}_{ig,1}^{\ast }={T}_{0}{(\frac{{\dot{m}^{\prime\prime} }_{cri}}{2\sqrt{\alpha {t}_{\max ,1}}\rho ZA{C}_{1}})}^{1/B}\approx {T}_{0}{(\frac{{\dot{m}^{\prime\prime} }_{cri}}{2\sqrt{20\alpha }\rho ZA{C}_{1}})}^{1/B}$$

where ${T}_{ig,1}^{\ast }$ is the equivalent ignition temperature under linearly decreasing HF. Equation (22) is an implicit expression because ${\theta }_{ig,1}^{\ast }$ actually is a function of *t*_max,1_. However, a characteristic value of *t*_max,1_ = 20 *s* is used in Eq. (23) to obtain an explicit result. When *t*_max,1_ changes from 10 s to 50 s, ${(1/\sqrt{{t}_{\max ,1}})}^{1/B}$ varies from 0.95 to 0.92 and this small discrepancy can be neglected. Solving Eq. (22), the ignition range of *a* can be calculated as:

24 $$a\le {a}_{cri,1}=\frac{8{\dot{q^{\prime\prime} }}_{0}^{3}}{9\pi k\rho {C}_{P}{({T}_{ig,1}^{\ast }-{T}_{0})}^{2}}$$

The ignition time at the critical condition can be expressed as:

25 $${t}_{ig,cri,1}=\frac{{\dot{q^{\prime\prime} }}_{0}}{2{a}_{cri,1}}=\frac{9\pi k\rho {C}_{P}{\theta }_{ig,1}^{\ast 2}}{16{\dot{q^{\prime\prime} }}_{0}^{2}}$$

If *a* is larger than *a*_*cri*,1_, no ignition is observed. When ${\dot{m}^{\prime\prime} }_{{\rm{\max }},1} > {\dot{m}^{\prime\prime} }_{cri}$, at ignition time we can get:

26 $$\frac{2\sqrt{{t}_{ig,1}}}{{T}_{0}\sqrt{\pi k\rho {C}_{P}}}({\dot{q^{\prime\prime} }}_{0}-\frac{2}{3}a{t}_{ig,1})={\theta }_{ig,1}^{\ast }$$

Consequently, the ignition time can be calculated based on Cardano’s formula for cubic equation:

27 $$\frac{1}{\sqrt{{t}_{ig,1}}}={[\frac{p}{6{\rm{\Delta }}}-\frac{\sqrt{3}i}{2}(\frac{p}{3{\rm{\Delta }}}+{\rm{\Delta }})-\frac{{\rm{\Delta }}}{2}]}^{-1}$$

28 $$p=-\,\frac{3{\dot{q^{\prime\prime} }}_{0}}{2a};\,q=\frac{3{\theta }_{ig,1}^{\ast }\sqrt{\pi k\rho {C}_{P}}}{4a};\,{\rm{\Delta }}=\sqrt[3]{\sqrt{{(\frac{q}{2})}^{2}+{(\frac{p}{3})}^{3}}-\frac{q}{2}}$$

Although Eq. (27) provides an exact solution for ignition time, it is a relatively complicated correlation. An approximate method is introduced here to simplify the ignition time expression. Noticing that when $a < {a}_{cri,1}$, the ignition time is slightly less than the value obtained under constant HF. A reasonable approximation is to substitute the *t*_*ig*,1_ in brackets of Eq. (26) for the average value of *t*_*ig*,*const*_ and *t*_*ig*,*cri*,1_. Combining Eq. (25) and the correlation under constant hear flux:

29 $$\frac{1}{\sqrt{{t}_{ig,const}}}=\frac{2{\dot{q^{\prime\prime} }}_{0}}{{\theta }_{ig,1}^{\ast }\sqrt{\pi k\rho {C}_{P}}}$$

Equation (26) can be approximately rewritten as:

30 $$\begin{array}{rcl}\frac{1}{\sqrt{{t}_{ig,1}}} & = & \frac{2{\dot{q^{\prime\prime} }}_{0}}{{\theta }_{ig}^{\ast }\sqrt{\pi k\rho {C}_{P}}}(1-\frac{2a}{3{\dot{q^{\prime\prime} }}_{0}}\frac{{t}_{ig,const}+{t}_{ig,cri,1}}{2})\\  & \approx  & \frac{1}{\sqrt{{t}_{ig,const}}}(1-\frac{13\pi k\rho {C}_{P}{\theta }_{ig}^{\ast 2}}{48{\dot{q^{\prime\prime} }}_{0}^{3}}a)\end{array}$$

### Quadratically decreasing heat flux

Noticing that

31 $${i}^{5}erfc(\xi )=\frac{4+9{\xi }^{2}+2{\xi }^{4}}{240\sqrt{\pi }}{e}^{-{\xi }^{2}}-\frac{1}{4}(\frac{\xi }{8}+\frac{{\xi }^{3}}{6}+\frac{{\xi }^{5}}{30})erfc(\xi )$$

Under quadratically decreasing HF, the mass flux can be expressed according to Eqs (7–9), (13) and (31):

32 $$\begin{array}{rcl}\dot{m}^{\prime\prime}  & = & \rho Z{\delta }_{c}A\,{\int }_{0}^{1}\,\{1+\frac{2{\dot{q^{\prime\prime} }}_{0}\sqrt{t}}{{T}_{0}\sqrt{k\rho {C}_{P}}}[\frac{1}{\sqrt{\pi }}{e}^{-{\xi }^{2}}-\xi erfc(\xi )]\\  &  & -\,\frac{8a{t}^{5/2}}{{T}_{0}\sqrt{k\rho {C}_{P}}}[\frac{4+9{\xi }^{2}+2{\xi }^{4}}{30\sqrt{\pi }}{e}^{-{\xi }^{2}}{-(\frac{\xi }{4}+\frac{{\xi }^{3}}{3}+\frac{{\xi }^{5}}{15})erfc(\xi )]\}}^{B}d\xi \end{array}$$

Another exponential approximation in ref.^19^ is employed to simplify Eq. (32):

33 $$\frac{4+9{\xi }^{2}+2{\xi }^{4}}{30\sqrt{\pi }}{e}^{-{\xi }^{2}}-(\frac{\xi }{4}+\frac{{\xi }^{3}}{3}+\frac{{\xi }^{5}}{15})erfc(\xi )\approx \frac{2}{15\sqrt{\pi }}{e}^{-3.9{\xi }^{1.1}}$$

Based on Eqs (16) and (33), Eq. (32) can be simplified as:

34 $$\dot{m}^{\prime\prime} \approx \rho Z{\delta }_{c}A\,{{\int }_{0}^{1}[1+\frac{2\sqrt{t}}{{T}_{0}\sqrt{\pi k\rho {C}_{P}}}({\dot{q^{\prime\prime} }}_{0}{e}^{-2.3{\xi }^{1.1}}-\frac{8}{15}a{t}^{2}{e}^{-3.9{\xi }^{1.1}})]}^{B}d\xi $$

Being similar to the simplification process from Eqs (18) to (19), another approximation is invoked to simplify Eq. (34):

35 $$\dot{m}^{\prime\prime} \approx \rho Z{\delta }_{c}A\,{{\int }_{0}^{1}\{[1+\frac{2\sqrt{t}}{{T}_{0}\sqrt{\pi k\rho {C}_{P}}}({\dot{q^{\prime\prime} }}_{0}-\frac{8}{15}a{t}^{2}){e}^{-0.85\xi }]\}}^{B}d\xi $$

**Figure 3:**
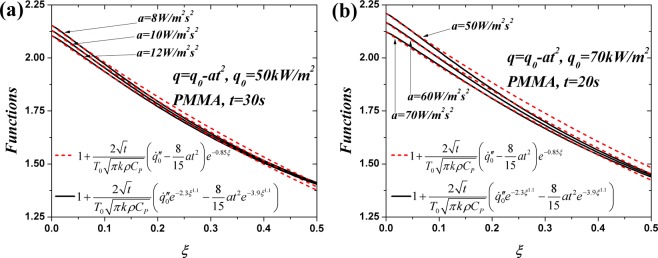
Comparison of the expressions in the square brackets of Eq. (34).

The reliability of this assumption is validated in Fig. 3 and the agreement is good especially in the vicinity of the high temperature surface. After integrating Eq. (35), the mass flux can be consequently expressed as:

36 $$\dot{m}^{\prime\prime} =\rho Z{\delta }_{c}A{C}_{2}{[1+\frac{2\sqrt{t}}{{T}_{0}\sqrt{\pi k\rho {C}_{P}}}({\dot{q^{\prime\prime} }}_{0}-\frac{8}{15}a{t}^{2})]}^{B}$$

37 $${C}_{2}=(1-{e}^{-0.85B})/0.85B$$

Similarly, the occurrence of ignition requires ${\dot{m}^{\prime\prime} }_{{\rm{\max }},2}\ge {\dot{m}^{\prime\prime} }_{cri}$ which results in the following expression through Eq. (36):

38 $$\frac{2\sqrt{{t}_{\max ,2}}}{{T}_{0}\sqrt{\pi k\rho {C}_{P}}}({\dot{q^{\prime\prime} }}_{0}-\frac{8}{15}a{t}_{\max \,,2}^{2})\ge {\theta }_{ig,2}^{\ast };\,{\theta }_{ig,2}^{\ast }={T}_{ig,2}^{\ast }-{T}_{0}$$

39 $${T}_{ig,2}^{\ast }={T}_{0}{(\frac{{\dot{m}^{\prime\prime} }_{cri}}{2\sqrt{\alpha {t}_{\max ,2}}\rho ZA{C}_{2}})}^{1/B}\approx {T}_{0}{(\frac{{\dot{m}^{\prime\prime} }_{cri}}{2\sqrt{20\alpha }\rho ZA{C}_{2}})}^{1/B}$$

The ignition range of *a* can be calculated as:

40 $$a\le {a}_{cri,2}=\frac{1536{\dot{q^{\prime\prime} }}_{0}^{5}}{625{(\pi k\rho {C}_{P})}^{2}{({T}_{ig,2}^{\ast }-{T}_{0})}^{4}}$$

Similar to Eq. (25), the ignition time at the critical condition can be expressed as:

41 $${t}_{ig,cri,2}=\frac{25\pi k\rho {C}_{P}{\theta }_{ig,2}^{\ast 2}}{64{\dot{q^{\prime\prime} }}_{0}^{2}}$$

If $a > {a}_{cri,2}$, no ignition takes place. When $a < {a}_{cri,2}$, at ignition time Eq. (36) can be written as:

42 $${\theta }_{ig,2}^{\ast }=\frac{2\sqrt{{t}_{ig,2}}}{\sqrt{\pi k\rho {C}_{P}}}({\dot{q^{\prime\prime} }}_{0}-\frac{8}{15}a{t}_{ig,2}^{2})$$

Similar to Eq. (30), the ignition time can be approximately solved as:

43 $$\frac{1}{\sqrt{{t}_{ig,2}}}\approx \frac{1}{\sqrt{{t}_{ig,const}}}(1-\frac{1681{(\pi k\rho {C}_{P})}^{2}{\theta }_{ig,2}^{\ast 4}}{30720{\dot{q^{\prime\prime} }}_{0}^{5}}a)$$

## Numerical Model

A previously developed numerical model^23^ was used to validate the established analytical model and experimental results. Both surface and in-depth absorptions and their combination were considered in the numerical model. The energy and mass conservation equations and decomposition rate in solid are:

44 $$\begin{array}{rcl}\rho {C}_{p}\frac{\partial T}{\partial t} & = & \frac{\partial }{\partial x}(k\frac{\partial T}{\partial x})+(1-r-\tau ){\dot{q}^{\prime\prime} }_{0}\kappa {e}^{-\kappa x}\\  &  & +\,\rho {S}_{v}[{\rm{\Delta }}{H}_{v}+(T-{T}_{0})({C}_{P}-{C}_{g})]-\dot{m}^{\prime\prime} {C}_{g}\frac{\partial {T}_{s}}{\partial x}\end{array}$$

45 $$\frac{\partial \rho }{\partial t}=\rho {S}_{v}$$

46 $${S}_{v}=-\,Z\cdot exp(\,-\,E/RT)$$

where *r* and $\tau$ denote the reflectivity, absorptivity of top surface, *S*_*v*_ is the rate of volatiles generation in solid, Δ*H*_*v*_ is the heat of decomposition, *C*_*g*_ is specific heat of gas, $\dot{m}^{\prime\prime}$ is the mass flux in the controlled volume, *Z* is pre-exponential factor, *E* is activation energy and *R* is ideal gas constant. $\tau =0$ denotes in-depth absorption and $\tau ={1}-r$ means surface absorption. When $\tau \in (0,1-r)$, both exist. The initial and boundary conditions are:

47 $$T(x,0)={T}_{0};\rho (x,0)={\rho }_{0}$$

48 $$\{\begin{array}{l}x=0:-\,k\frac{\partial T}{\partial x}=\tau {\dot{q}^{\prime\prime} }_{0}-\varepsilon \sigma ({T}^{4}-{T}_{0}^{4})-{h}_{c}(T-{T}_{0})\\ x=L:{-\frac{\partial T}{\partial x}|}_{x=L}=0\end{array}$$

where *L* denotes the thickness of sample. Although temperature-dependent thermal parameters were considered in this numerical model, constant values at ambient temperature were used for comparison with analytical model. More detailed information about the numerical model can be found in ref.^23^. The simulation time, spatial and time steps are 500 s, 0.05 mm and 0.2 s, respectively. In order to verify the thermally thick assumption, 30 and 50 mm thick sample cases were also simulated. Little discrepancy of the in-depth temperature was found between the two cases. Therefore, 30 mm thickness was used in this study to save the simulation time.

## Results and Discussion

PMMA is selected for computation to validate the proposed analytical model in this section. The thermophysics, chemical kinetics and other relevant parameters of PMMA are listed in Table 1.

**Table 1:** Parameters of black PMMA (Polymethyl Methacrylate) used in calculation.

Parameters	PMMA	
	Values	Ref.
Density, *ρ* (kg/m^3^)	1190	^48^
Specific heat, *C*_*P*_ (J/(gK))	1.7	^48^
Thermal conductivity, *k* (J/(smK))	0.336	^48^
Pre-exponential factor, Z (1/s)	5 × 10^8^	^48^
Activation temperature, *T*_*a*_ (K)	1.5 × 10^4^	^48^
Ambient temperature, *T*_*∞*_ (K)	300	—
Critical mass flux, $\dot{m}^{\prime\prime}$(g/(m^2^ s))	2.42	^3^
*A*	5.654 × 10^−19^	^8^
*B*	24.39	^8^
*T*_*1*_ (K)	357	^8^
*T*_*2*_ (K)	615	^8^

### Surface temperature

The exact solutions of surface temperature can be expressed based on Eqs (7), (13), (14) and (31) for linearly and quadratically decreasing HFs:

49 $$\theta (0,t)=\frac{2\sqrt{t}}{\sqrt{\pi k\rho {C}_{P}}}({\dot{q^{\prime\prime} }}_{0}-\frac{2}{3}at)$$

50 $$\theta (0,t)=\frac{2\sqrt{t}}{\sqrt{\pi k\rho {C}_{P}}}({\dot{q^{\prime\prime} }}_{0}-\frac{8}{15}a{t}^{2})$$

**Figure 4:**
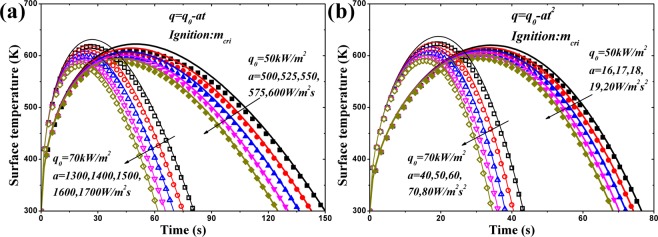
Comparison of surface temperature of PMMA between analytical and numerical models: (**a**) Linearly decreasing HF; (**b**) Quadratically decreasing HF. Solid symbols: numerical results at ${\dot{q^{\prime\prime} }}_{0}=50\,kW/{m}^{2}$; Hollow symbols: numerical results at ${\dot{q^{\prime\prime} }}_{0}=70\,kW/{m}^{2}$; Thick solid curves: analytical results at ${\dot{q^{\prime\prime} }}_{0}=50\,kW/{m}^{2}$; Thin solid curves: analytical results at ${\dot{q^{\prime\prime} }}_{0}=70\,kW/{m}^{2}$. The value of *a* increases in the direction of the arrow.

The comparison of surface temperature of PMMA between the developed analytical model and simulation results is illustrated in Fig. 4. *a*_*cri*_ is crucial for ignition and the ranges of *a* used in Fig. 4 are in the vicinity of *a*_*cri*_. The calculated values of *a*_*cri*_ through critical mass loss rate under the initial HFs of 50 and 70 kW/m^2^ are listed in Table 2. For fixed ${\dot{m}^{\prime\prime} }_{cri}$, *a*_*cri*,1_ and *a*_*cri*,2_ are proportional to ${\dot{q^{\prime\prime} }}_{0}^{3}$ and ${\dot{q^{\prime\prime} }}_{0}^{5}$, respectively, based on Eqs (24) and (40), and thus *a*_*cri*_ increases with increasing initial HF. When $a\le {a}_{cri}$, ignition occurs in air and the established flame would affect the surface temperature. However, the purpose of this section is to verify the capability of the analytical model. Therefore, the heating is assumed to be conducted in anaerobic environment and no ignition is examined in Fig. 4.

**Table 2:** Calculated *a*_*cri*_ of PMMA by critical mass flux under different HFs.

${\dot{{\boldsymbol{q}}}{\boldsymbol{^{\prime\prime} }}}_{{\boldsymbol{0}}}({\boldsymbol{k}}{\boldsymbol{W}}{\boldsymbol{/}}{{\boldsymbol{m}}}^{{\boldsymbol{2}}})$	*a*_*cri*_ calculated by ${\dot{{\boldsymbol{m}}}^{\prime\prime} }_{{\boldsymbol{c}}{\boldsymbol{r}}{\boldsymbol{i}}}$	
	$\dot{{\boldsymbol{q}}}{\boldsymbol{^{\prime\prime} }}{\boldsymbol{=}}{\dot{{\boldsymbol{q}}}{\boldsymbol{^{\prime\prime} }}}_{{\boldsymbol{0}}}{\boldsymbol{-}}{\boldsymbol{a}}{\boldsymbol{t}}$	$\dot{{\boldsymbol{q}}}{\boldsymbol{^{\prime\prime} }}{\boldsymbol{=}}{\dot{{\boldsymbol{q}}}{\boldsymbol{^{\prime\prime} }}}_{{\boldsymbol{0}}}{\boldsymbol{-}}{\boldsymbol{a}}{{\boldsymbol{t}}}^{{\boldsymbol{2}}}$
50	508.13	17.11
70	1394.30	92.00

*The units of *a*_*cri*_ are Wm^−2^ s^−1^ and Wm^−2^ s^−2^ for linearly and quadratically decreasing HFs, respectively.

As expected, the surface temperature gets higher as *a* decreases. When pyrolysis is considered in numerical model. The exact analytical solutions fit the simulation results relatively well, but still some discrepancy is observed in Fig. 4. Especially in the range near the peak value, the analytical model deviates from the numerical results most. This divergence is caused by the gasification heat absorbed during pyrolysis in the numerical model. A small portion of the absorbed energy is consumed in the thermal degradation to evaporate the condensed solid. However, in the analytical model all the energy is assumed to heat the thermally inert material. Consequently, the analytical curves are slightly higher than the numerical ones. In the early and late stages, the lower surface temperature leading to a weaker pyrolysis rate results in a better agreement. The maximum discrepancy of surface temperature caused by the thermal degradation in Fig. 4 between the analytical and numerical models is less than 20 K.

### Transient mass flux

**Figure 5:**
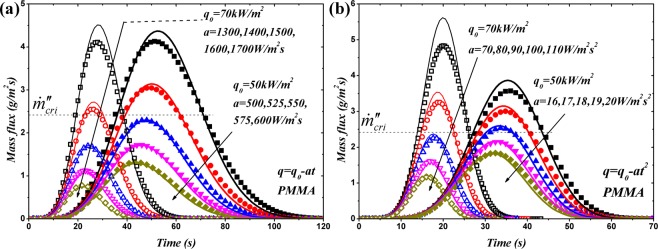
Comparison of transient mass flux of PMMA between analytical and numerical models: (**a**) Linearly decreasing HF; (**b**) Quadratically decreasing HF. Solid symbols: numerical results at ${\dot{q^{\prime\prime} }}_{0}=50\,kW/{m}^{2}$; Hollow symbols: numerical results at ${\dot{q^{\prime\prime} }}_{0}=70\,kW/{m}^{2}$; Thick solid curves: analytical results at ${\dot{q^{\prime\prime} }}_{0}=50\,kW/{m}^{2}$; Thin solid curves: analytical results at ${\dot{q^{\prime\prime} }}_{0}=70\,kW/{m}^{2}$. The value of *a* increases in the direction of the arrow.

Transient mass flux reflects the generation rate of volatiles and determines the subsequent ignition. Figure 5 demonstrates the comparison of mass flux of PMMA under linearly and quadratically decreasing HFs without considering the ignition effect. Also, *a*_*cri*_ listed in Table 1 can be calculated through Eqs (25) and (41). The value of *a* used in analytical and numerical models in Fig. 5 spans the ignition and no-ignition domains. The resultant peak values higher and lower than the critical mass flux, 2.42 g/m^2^ s, imply the ignition and no-ignition scenarios, respectively. The developed analytical model fits the simulation results reasonably well except the peak values in low *a* cases. This disagreement is also attributed to the temperature related pyrolysis rate. Higher temperature resulting from lower *a* vaule would enhance the thermal degradation and consume more absorbed energy during the gasification. While with higher *a* values, the agreement is good. Although the disagreement of peak values is significant in the ignition cases, the prediction of ignition time is barely affected since the agreement at critical mass flux, 2.42 g/m^2^ s, is good during the increasing phase of the curves in Fig. 5. Predictably, this distinction of peak value would induce the maximal error of ignition time in the critical case, *a* = *a*_*cri*_, which will also be demonstrated later.

### Ignition time

**Figure 6:**
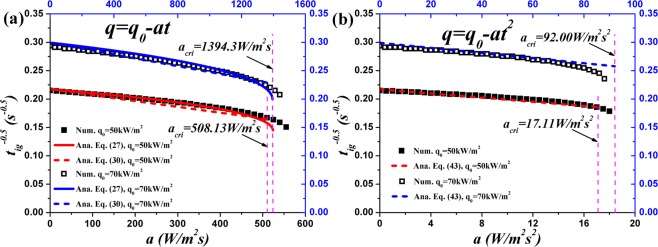
Comparison of ignition time of PMMA between analytical and numerical models.

Figure 6 shows the predicted ignition time of PMMA under linearly and quadratically decreasing HFs through analytical and numerical models. Under linearly decreasing HF, Fig. 6 (a), Eq. (27) provides good agreement with the numerical simulation by solving the cubic equation even though several approximations are invoked, including Eqs (9), (16), (17), (19) and (23). Without solving the cubic and quintic equations, the approximate analytical solutions, Eqs (30) and (43), provide linear correlations between ${t}_{ig}^{-0.5}$ and *a* for both linearly and quadratically decreasing HFs. In Fig. 6, the straight lines agree well with the numerical simulation until *a*_*cri*_. When *a* approaches *a*_*cri*_, ${t}_{ig}^{-0.5}$ decreases sharply. However, the ignition time at *a*_*cri*_ can be attained through Eqs (25) and (41).

## Parametric Study of Critical Mass Flux

Critical mass flux is a determinant input parameter in analytical and numerical ignition models for specified materials. The objective of this section is to investigate the influence of ignition criteria on ignition time.

Although the critical mass flux of PMMA used in Section 3.3 is 2.42 g/m^2^ s, it is hard to be measured accurately in the tests and it is sensitive to the experimental condition. Therefore, utilization of a valid range of this parameter may be a more practicable choice. The reported critical mass flux ranges of PMMA by Bal^3^ and Vermesi^12^ are 1.82–3.75 and 1.9–3.2 g/m^2^ s, respectively. However, a larger range, 1–8 g/m^2^ s is used in this study to examine its influence on ignition time. The equivalent ignition temperatures of PMMA, ${T}_{ig}^{\ast }$, are calculated through Eqs (23) and (39), and the values are listed in Table 3 for the focused two types of HFs. ${T}_{ig}^{\ast }$ is independent of ${\dot{q^{\prime\prime} }}_{0}$ and increases with ${\dot{m}^{\prime\prime} }_{cri}$ because it is proportional to ${\dot{m}}_{cri}^{^{\prime\prime} 1/B}$ in Eqs (23) and (39). All these values are lower than *T*_*ig*_, 655.1 K. Furthermore, the values of *a*_*cri*_ of PMMA computed through Eqs (24) and (40) using critical mass flux are given in Table 4. Not surprisingly, for fixed initial HF *a*_*cri*_ decreases with increasing ${\dot{m}^{\prime\prime} }_{cri}$, indicating that lower *a*_*cri*_ is prerequisite to heat the solid to higher temperature and generate more volatiles. *a*_*cri*_ also increases with initial HF because ${a}_{cri,1}\propto {\dot{q}}_{0}^{^{\prime\prime} 3}$ and ${a}_{cri,2}\propto {\dot{q}}_{0}^{^{\prime\prime} 5}$ for linearly and quadratically decreasing HFs, respectively.

**Table 3:** Calculated ${T}_{ig}^{\ast }$ of PMMA by different critical mass fluxes under different initial HFs.

*m*_*cri*_ (g/m^2^ s)	${{\boldsymbol{T}}}_{{\boldsymbol{i}}{\boldsymbol{g}}}^{\ast }$ (K)	
	$\dot{{\boldsymbol{q}}}{\boldsymbol{^{\prime\prime} }}{\boldsymbol{=}}{\dot{{\boldsymbol{q}}}{\boldsymbol{^{\prime\prime} }}}_{{\boldsymbol{0}}}{\boldsymbol{-}}{\boldsymbol{a}}{\boldsymbol{t}}$	$\dot{{\boldsymbol{q}}}{\boldsymbol{^{\prime\prime} }}{\boldsymbol{=}}{\dot{{\boldsymbol{q}}}{\boldsymbol{^{\prime\prime} }}}_{{\boldsymbol{0}}}{\boldsymbol{-}}{\boldsymbol{a}}{{\boldsymbol{t}}}^{{\boldsymbol{2}}}$
1	597.55	593.58
2.42	619.60	615.48
8	650.73	646.41

**Table 4:** Calculated *a*_*cri*_ of PMMA by different critical mass fluxes under different initial HFs.

*m*_*cri*_ (g/m^2^ s)	$\dot{{\boldsymbol{q}}}{\boldsymbol{^{\prime\prime} }}{\boldsymbol{=}}{\dot{{\boldsymbol{q}}}{\boldsymbol{^{\prime\prime} }}}_{{\boldsymbol{0}}}{\boldsymbol{-}}{\boldsymbol{a}}{\boldsymbol{t}}$		$\dot{{\boldsymbol{q}}}{\boldsymbol{^{\prime\prime} }}{\boldsymbol{=}}{\dot{{\boldsymbol{q}}}{\boldsymbol{^{\prime\prime} }}}_{{\boldsymbol{0}}}{\boldsymbol{-}}{\boldsymbol{a}}{{\boldsymbol{t}}}^{{\boldsymbol{2}}}$	
	${\dot{{\boldsymbol{q}}}{\boldsymbol{^{\prime\prime} }}}_{{\boldsymbol{0}}}{\boldsymbol{=}}{\boldsymbol{50}}\,{\boldsymbol{k}}{\boldsymbol{W}}{\boldsymbol{/}}{{\boldsymbol{m}}}^{{\boldsymbol{2}}}$	${\dot{{\boldsymbol{q}}}{\boldsymbol{^{\prime\prime} }}}_{{\boldsymbol{0}}}{\boldsymbol{=}}{\boldsymbol{70}}\,{\boldsymbol{k}}{\boldsymbol{W}}{\boldsymbol{/}}{{\boldsymbol{m}}}^{{\boldsymbol{2}}}$	${\dot{{\boldsymbol{q}}}{\boldsymbol{^{\prime\prime} }}}_{{\boldsymbol{0}}}{\boldsymbol{=}}{\boldsymbol{50}}\,{\boldsymbol{k}}{\boldsymbol{W}}{\boldsymbol{/}}{{\boldsymbol{m}}}^{{\boldsymbol{2}}}$	${\dot{{\boldsymbol{q}}}{\boldsymbol{^{\prime\prime} }}}_{{\boldsymbol{0}}}{\boldsymbol{=}}{\boldsymbol{70}}\,{\boldsymbol{k}}{\boldsymbol{W}}{\boldsymbol{/}}{{\boldsymbol{m}}}^{{\boldsymbol{2}}}$
1	587.70	1612.60	22.67	121.93
2.42	509.40	1397.80	17.00	91.44
8	422.99	1160.70	11.70	62.90

*The units of *a*_*cri*_ are Wm^−2^ s^−1^ and Wm^−2^ s^−2^ for linearly and quadratically decreasing HFs, respectively.

**Figure 7:**
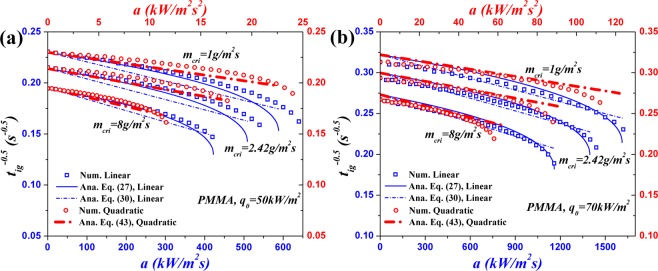
Effect of critical mass flux on ${t}_{ig}^{-0.5}$ of PMMA: (**a**) ${\dot{q^{\prime\prime} }}_{0}=50\,kW/{m}^{2}$; (**b**) ${\dot{q^{\prime\prime} }}_{0}=70\,kW/{m}^{2}$.

Figure 7 shows the influence of ${\dot{m}^{\prime\prime} }_{cri}$ on ${t}_{ig}^{-0.5}$ of PMMA with initial HFs of 50 and 70 kW/m^2^. The agreement between analytical and numerical results in this figure is not very good but acceptable because several additional approximations are employed when considering thermal degradation reaction within solid. As *a* getting closed to *a*_*cri*_, the discrepancy gets larger. This is caused by the fact that the induced error by invoking the approximations approaches a climax when *a* = *a*_*cri*_, which has been interpreted in the end of Section 3.2. As shown in Fig. 7, the critical mass flux has significant effect on the ignition time.

## Conclusions

An approximate analytical model is established in this study to investigate the ignition of solids exposed to a time-decreasing incident HF. Critical mass flux is used in the model to calculate the surface temperature, transient mass flux and ignition time based on the exactly obtained in-depth temperature. Linearly and quadratically decreasing HFs are focused. An approximation methodology is proposed to simplify the complicated expressions during the derivation. The reliability of the developed model is verified by comparison with a numerical model employing PMMA as the reference material.

For time-decreasing HF, $\dot{q^{\prime\prime} }={\dot{q^{\prime\prime} }}_{0}-a{t}^{b}$, *a* must be lower than a critical value, *a*_*cri*_, to ensure the ignition occurrence. This *a*_*cri*_ is proportional to ${\dot{q}}_{0}^{^{\prime\prime} 3}$ and ${\dot{q}}_{0}^{^{\prime\prime} 5}$ for linearly and quadratically decreasing HFs, respectively. A linear dependence is found between ${t}_{ig}^{-0.5}$ and $a$. Also, ${t}_{ig}^{-0.5}$ under time-decreasing HF is correlated with the ignition time under constant HF. An equivalent ignition temperature, ${T}_{ig}^{\ast }$, which is identical to *T*_*ig*_, is found to keep the ignition time expression similar to the one in critical temperature case. ${T}_{ig}^{\ast }$ is independent of ${\dot{q^{\prime\prime} }}_{0}$ but is proportional to ${\dot{m}}_{cri}^{^{\prime\prime} 1/B}$. The accuracy of the proposed model is good for high values of *a* and decreases as *a* gets lower when predicting the transient mass flux. However, this increased inaccuracy does not compromise the capability of ignition time prediction. The error induced by invoking the approximations gets larger as *a* approaching *a*_*cri*_. According to the parametric study, critical mass flux has great effect on the ignition time prediction.

Although only linearly and quadratically decreasing HFs are studied in this work, the derivation procedure and the approximation method can be extended to other time-decreasing HF scenarios. The main criticism of this work is the negligence of the surface heat loss. The temperature-dependent heat loss in boundary condition would greatly complicate the derivation, and thus more future studies are needed to solve this issue. Meanwhile, in-depth absorption of thermal radiation within infrared translucent solids and the corresponding heat transfer and ignition problems also deserve more attention.
